# Cardiovascular magnetics resonance diagnosis of cystic tumor of the atrioventricular node

**DOI:** 10.1186/1532-429X-11-13

**Published:** 2009-04-30

**Authors:** Thao T Tran, Vaughn Starnes, Xuedong Wang, James Getzen, Brian D Ross

**Affiliations:** 1 Huntington Medical Research Institutes, Pasadena, CA, USA; 2 LA County Hospital, LA, CA, USA; 3 Huntington Memorial Hospital, Pasadena, CA, USA

## Abstract

Late gadolinium enhanced (LGE) cardiovascular magnetic resonance (CMR) has proven to be the gold standard for viability assessment. LGE CMR is also useful for identifying the nature of cardiac masses or lesions. We report a case of a rare primary cystic tumor of the atrioventricular node, in which CMR proved to be valuable.

## Background

Although primary cystic tumor of the atrioventricular node is very rare and classified as benign [1], it may lead to significant morbidity and mortality due to obstruction, arrhythmias and embolism [2]. Cystic tumors usually consist of multi-cystic nodules and are located in the region of the atrioventricular (AV) node [2]. It is rarely found in the literature[3]. This is a difficult tumor to identify on non-invasive studies or clinical findings, especially to distinguish from myxomas. Treatment requires immediate surgery. We have identified three [4-6] previous surgical successes with this otherwise almost invariably fatal cardiac tumor. The use of cardiovascular magnetic resonance (CMR) and computed tomography (CT) have been helpful in identifying tumors occurring in the intra-atrial septum and the AV node. Paniaga et al [4] included an axial T1 weighted CMR, which is similar to the current case but gives no information about LGE. The other [5] gives no CMR information.

## Case presentation

The patient, a 42-year old female, was referred for CMR with suspected atrial myxoma and the confirmation was needed for pre-surgical evaluation. She presented with episodes of syncope, mitral valve prolapse and suspected atrial myxoma. She had four successful pregnancies, however, developed shortness of breath about 1 month after the birth of her fourth infant. An electrocardiogram (ECG) was performed with results indicating sinus rhythm with first degree AV block and PR interval of nearly 400 ms, an LV ejection fraction (EF) of 50%, mildly dilated left ventricle (LV = 58 mm and 42 mm), left atrial enlargement and mitral regurgitation. Two weeks after the ECG study, the patient was admitted to the hospital due to recurrent syncope and a repeated ECG showed abnormal AV node function and the subsequently had a dual chamber pacemaker implanted. About a month after the implantation, the patient had persistent swelling and yellowish discharge due to infection in the peripherally inserted central catheter (PICC) line and the pacemaker had to be removed. An echocardiogram revealed a myxomatous mass in the right atrium. The patient was then scheduled for surgery to remove the mass, with CMR preceding the surgery for confirmation of the right atrial myxoma [7].

The patient was scanned in a 1.5 T clinical scanner (General Electric, Milwaukee, WI) using a 4-element phased array coil and gated by a 4-lead electrocardiogram (ECG). Two doses of gadopentetate dimeglumine (Magnevist, Berlex, Wayne, NJ) were injected (the first, 0.05 mL/kg at 2 mL/second for perfusion imaging and the second, 0.1 mL/kg for late gadolinium enhancement) using a Spectrus power injector (Medrad, Indianola, PA). The protocol used is as follows: (1) sagittal localizer using fast spoiled grass (fSPGR); (2) long axis localizer using Fast Imaging Employing Steady State Acquisition (FIESTA); (3) short axis using FIESTA; (4) perfusion imaging using fast gradient echo-echo train (FGRE-ET); (5) cine radial views using FIESTA; (6) Late Gadolinium Enhancement (LGE) approximately 10 minutes post-intravenous injection. All acquisitions, except for the perfusion study, were performed with breath-holding and the entire exam was accomplished in 45 minutes. The data was analyzed and quantified using Advantage Workstation (GE LX, Milwaukee, WI) and MASS Analysis Plus software (MEDIS, Netherlands).

**Figure 1:**
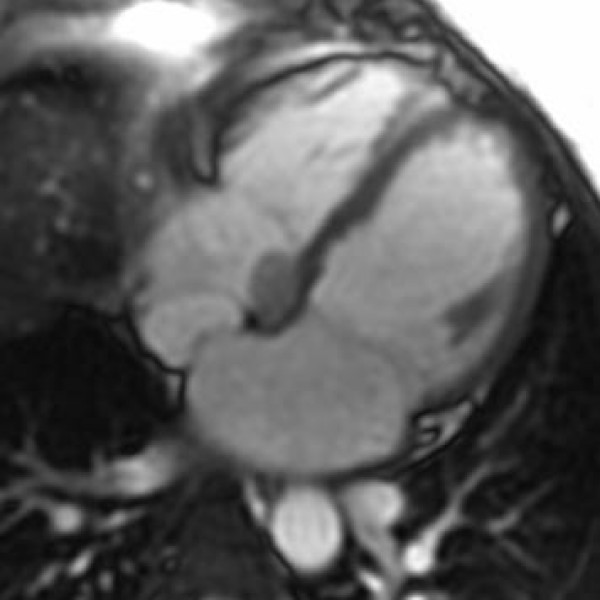
**Four chamber long axis view revealing the tumor at the atrioventricular node**.

**Figure 2:**
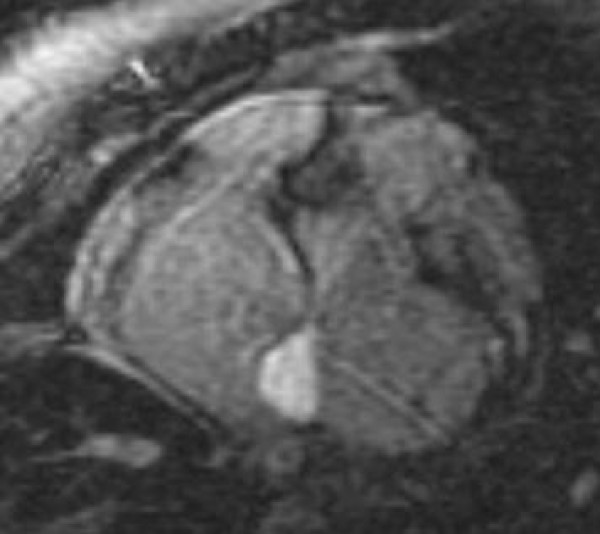
**Late gadolinium enhancement of the tumor**.

**Figure 3:**
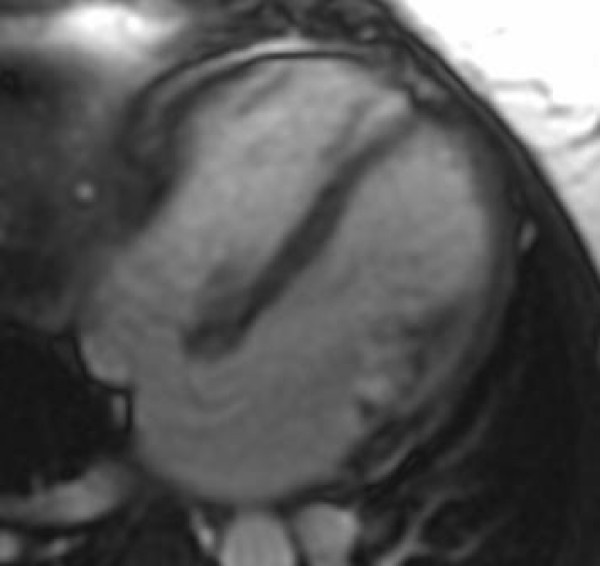
**Four chamber long axis view demonstrating an atrial-septal defect with left-to-right shunting**.

**Figure 4:**
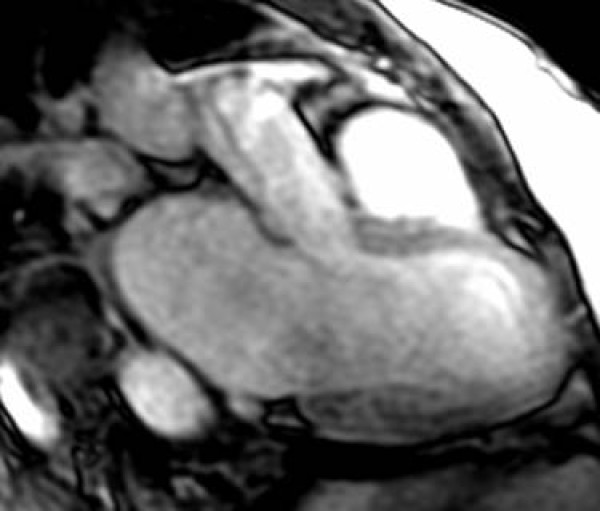
**Three chamber view of the left atrium, left ventricle and right ventricle demonstrating modest mitral incompetence and large left atrium**.

The standard imaging sequences revealed a hypointense cardiac mass (Figure 1), measuring 2.5 × 1.5 cm^2^, located in the right atrium (RA) on the atrial-septal wall immediately above the medial leaflet of the tricuspid valve. Furthermore, there were no changes in the size of the mass during systole and diastole phases. LGE revealed an-enhancing mass (Figure 2), consistent with a myxoma. There is also an atrial septal defect (ASD) (Figure 3) with left to right shunting which presented as two "jets" superior and inferior to the mass. A very modest mitral regurgitant jet was noted (Figure 4), with significant left atrial enlargement and slight increase in end diastolic LV volume (EDV = 134 ml; normal = 98–127). The patient's ejection fraction (EF = 60%), stroke volume (SV = 80 ml) and cardiac index (CI = 2.5 L/min/m2) were in the normal range. Differential diagnosis included right atrial myxoma due to isointensity on FSPGR images, hyperintensity on LGE images with heterogeneous contrast enhancement [8]. However, the mass was 2.5 cm in diameter, compared to myxomas which are usually 6 cm in size, and was hypointense on FIESTA images. Combining this information with the histopathology report, the final diagnosis is a right atrial cystic tumor of the AV node.

A surgical sternotomy and excision of the atrial tumor was performed and findings include an atrial-septal defect and a golf ball size tumor measuring 2 × 3 cm^2 ^between the coronary sinus and the tricuspid valve annulus. The tricuspid valve was preserved and the roof of the coronary sinus was protected. The surgical area was patched using pericardial patch and the atrial septal defect was also closed. It was noted that the patient was in heart block postoperatively. A few days following the resection, the patient received a new dual chamber pacemaker. The patient made a full and uneventful recovery.

**Figure 5:**
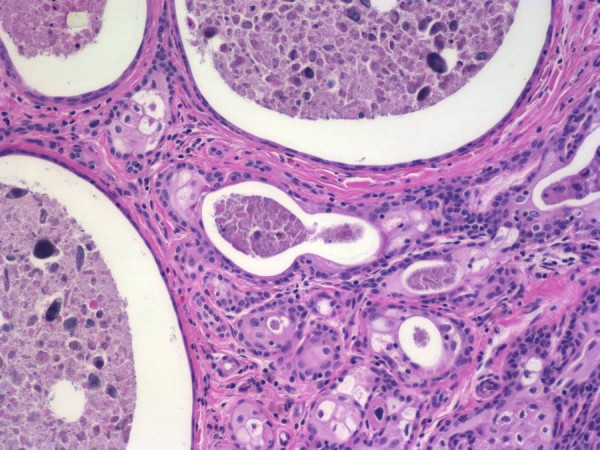
**Histology image of the tumor shows cysts of variable sizes filled with keratinous debris and nests of epithelial cells with squamous differentiation**.

**Figure 6:**
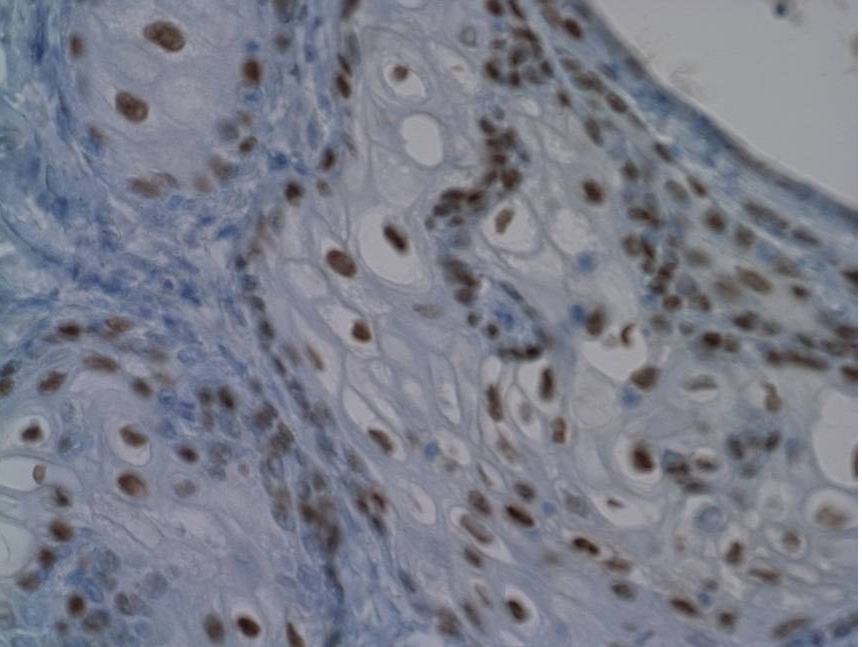
**The tumor was strongly positive for estrogen and focally positive for progesterone receptor**.

A sample of the tumor was sent for pathology investigation. The specimen had a large amount of orange/brown thickened debris which was then sent to microbiology for aerobic, bacterial and fungal studies. The histology report indicated that the tumor was filled with abundant brown amorphous material. It consisted of multiple cysts (Figure 5) of variable sizes and nests of cells with a fibrotic background interspersed with chronic inflammatory cells. The cysts were lined with squamous cells with abundant keratinous debris within the cysts. The morphologic appearance was consistent with cystic tumor of the atrioventricular node. Since the patient developed symptoms during her pregnancy, immunostains for estrogen and progesterone receptors (Figure 6) were also performed. This was strongly positive for estrogen receptors and focally positive for progesterone receptors, indicating that the tumor might have been progressive during the pregnancy stage due to increase levels of hormones. A study by Letterie et al [9] found a benign cystic atrio-ventricular nodal tumor treated with a long-acting gonadotropin releasing hormone (GnRH) decreased in size. However, with the addition of a combination of estrogen and progestin, the cyst gradually increased in size and progressed even after the discontinuation of all therapy. This study and our findings suggest that there might be a correlation between the progression of such tumors and the levels of hormones present.

## Consent

Written informed consent was obtained from the patient for publication of this case report and any accompanying images.

## Competing interests

The authors declare that they have no competing interests.

## Authors' contributions

TT acquired the MR images and carried out data analysis. VS is the surgeon responsible for removing the tumor. XW performed and analyzed the histopathology of the tumor. JG and BDR aided in the analysis of the MRI and the diagnosis of the tumor. All authors read and approved the final manuscript.
